# Nanophyto-gel against multi-drug resistant *Pseudomonas aeruginosa* burn wound infection

**DOI:** 10.1080/10717544.2021.1889720

**Published:** 2021-02-23

**Authors:** Ming Ming Wen, Ibrahim A. Abdelwahab, Rania G. Aly, Sally A. El-Zahaby

**Affiliations:** a Department of Pharmaceutics & Pharmaceutical Technology, Faculty of Pharmacy, Pharos University in Alexandria, Alexandria, Egypt; b Department of Microbiology and Immunology, Faculty of Pharmacy, Pharos University in Alexandria, Alexandria, Egypt; c Department of Pathology, Faculty of Medicine, Alexandria University, Alexandria, Egypt

**Keywords:** Burn wounds, cinnamon oil, *Pseudomonas aeruginosa*, nanostructured lipid carriers, nanophyto-gel, phytotherapy

## Abstract

Burn wound is usually associated by antibiotic-resistant *Pseudomonas aeruginosa* infection that worsens and complicates its management. An effective approach is to use natural antibiotics such as cinnamon oil as a powerful alternative. This study aims to investigate topical nanostructured lipid carrier (NLC) gel loaded cinnamon oil for *Pseudomonas aeruginosa* wound infection. A 2^4^ full factorial design was performed to optimize the formulation with particle size 108.48 ± 6.35 nm, zeta potential −37.36 ± 4.01 mV, and EE% 95.39 ± 0.82%. FTIR analysis revealed no excipient interaction. Poloxamer 407 in a concentration 20% w/w NLC gel was prepared for topical application. Drug release exhibited an initial burst release in the first five hours, followed by a slow, sustained release of up to five days. NLC-cinnamon gel has a significant ability to control the drug release with the lowest minimum inhibitory concentration again *P. aeruginosa* compared to other formulations (*p* < .05). *In vivo* study also showed NLC-cinnamon gel effectively healed the infected burned wound after a six-day treatment course with better antibacterial efficacy in burned animal models. Histological examination ensured the tolerability of NLC-cinnamon gel. The results suggest that nanoparticle-based cinnamon oil gel is a promising natural product against antibiotic-resistant strains of *P. aeruginosa* in wound infection.

## 1. Introduction

Wound infection is the main risk factor in patients with burns, and burn wound caused by *Pseudomonas aeruginosa* is one of the most challenged wound care management in the hospitals (Jault et al., 2018). Difficulties in management may arise from the severe inflammation of the tissues and untreated infections as well as the biofilms that might be formed as a result of microbial infection introducing more resistance to cure. Without proper treatment, an acute wound could develop into a chronic wound due to the presence of more virulent bacterial species which increases the mobility and economic burden of patients (Rahim et al., 2017). Therefore, infection research is given as the top priority by the British Burns Association (BBA) for the patient benefit (The British Burns Association Research Special Interest Group, 2018).

There is a growing interest in the clinical use of natural products against antibiotic-resistant pathogens such as *Pseudomonas aeruginosa*, a biofilm-forming bacterium due to overuse of antibiotics. In this concern, more attention was given to include natural products in wound management seeking reduction of side effects encountered from too much consumption of chemicals, especially that the infected patients already had a special skin situation. One of these trending phyto-compounds is cinnamon bark oil which has been used in antimicrobial, antidiabetic, antioxidant, and anti-inflammatory applications with substantial beneficial evidence since ancient times. The active constituents of cinnamon oil are cinnamaldehyde (65–80%) and eugenol (5–10%) that have proven inhibition ability against *P. aeruginosa* to form antibiotic-resistant biofilms (Utchariyakiat et al., 2016). Another important feature of cinnamon oil is its ability to advance tissue regeneration and accelerate wound healing which will help wound patients in recovering their skin integrity (Seyed Ahmadi et al., 2019). However, low solubility and instability are the major limitation factors to achieve sustained delivery of this phyto-compound for clinical use (Bilia et al., 2014). There were only a few published topical preparations aiming to deliver cinnamon oil in nanocarriers such as nano cream (Zainol et al., 2015) and nanoemulsion (Mukerjee et al., 2019) and yet none addressed wound infection. Thus, it is essential to design a cinnamon oil delivery system to overcome these challenges to maximize its therapeutic efficiency.

A nanostructured lipid carrier (NLC) is a colloidal, nanoparticle system that composes of a mixture of solid and liquid lipids and stabilized by surfactants in an aqueous phase for drug delivery. It can entrap a large number of lipophilic agents due to the imperfect matrix arisen from the mixed lipid components which prevent homogeneous crystal formation (Beloqui et al., 2016). NLCs are highly biodegradable and biocompatible and provide better drug delivery and skin permeation compared to solid lipid nanoparticles (Garcês et al., 2018). NLCs have been demonstrated in delivering highly lipophilic drugs into targeted organs, and damaged tissues such as burn wounds (Zahir-Jouzdani et al., 2019). This study aims to investigate nanotechnology-based topical NLC-Cinnamon oil gel as a novel application for *P. aeruginosa* wound infection with better antibacterial efficacy *in vivo*.

## 2. Materials and methods

### 2.1. Materials

Stearic acid was purchased from BDH Laboratory (Poole, UK). Tween 80 and Poloxamer 407 were from Sigma-Aldrich Co. (St. Louis, MO). Precirol^®^ ATO 5 and Labrafac™ lipophile WL 1349 were kind gifts from Gattefosse (Lyon, France). All other chemicals used were of pharmaceutical grade.

### 2.2. Extraction of *Cinnamomum zeylanicum* bark oil

The Clevenger hydrodistillation method (Pharmac Engineers, Mumbai, India) was used to extract freshly prepared essential oil (EO) from cinnamon bark of *Cinnamomum zeylanicum* imported from Oman. The dried bark 400 g was first grounded into powder and transferred to a round bottom flask which was connected to the condenser and then boiled with one liter of distilled water for one hour and a half. The yield EO was dried over anhydrous sodium sulfate and stored in a dark glass bottle at 4–8 °C to prevent oxidative degradation. The final concentration used was 2.5 mg/ml. The extracted oil was analyzed using GC/MS (Thermo Fisher Scientific, Dreieich, Germany) (data not shown). The major volatile oil constituents included cinnamaldehyde, cinnamic acid, cinnamyl acetate, and eugenol.

### 2.3. Preparation of NSC-cinnamon oil

Two novel excipients Precirol ATO 5 and Labrafac™ lipophile WL 1349 were used to advance the formulation carrier. Labrafac is a mixture of medium-chain triglyceride and it is derived from vegetal origin and liquid at room temperature (Nanoscale, 2018). NSC-cinnamon oil was prepared according to the method described by Teng et al. (2019) with modification of temperature being 60 °C for both lipid and aqueous phases which were then stirred vigorously with a high-speed homogenizer (IKA T25, Digital ULTRA TURRAX^®^, Königswinter, Germany) at 20,000 rpm for 10 min and followed by sonification in a water bath sonicator for 5 min at 1500 W (Shyam Industries, Ahmedabad, India). The prepared NSCs were further continuously stirred at 100 rpm overnight at room temperature. The formulation compositions were presented in Table 1.

**Table 1.** Compositions of NLC formulations.

Formula	Precirol (mg)	Stearic acid (mg)	Labrafac (mg)	Cinnamon oil (mg)	Tween 80 (mg)	Water (g)
1	833	0	0	167	300	8.7
2	1667	0	166	167	50	8
3	500	0	333	167	300	8.7
4	1667	0	166	167	300	7.7
5	500	0	333	167	50	9
6	1000	0	833	167	50	8
7	1000	0	833	167	300	7.7
8	833	0	0	167	50	9
9	0	500	333	167	300	8.7
10	0	1667	166	167	300	7.7
11	0	1000	833	167	50	8
12	0	500	333	167	50	9
13	0	833	0	167	300	8.7
14	0	1000	833	167	300	7.7
15	0	1667	166	167	50	8
16	0	833	0	167	50	9

### 2.4. Factorial design for formulation optimization

A 2^4^ full factorial experimental design was used to investigate the main effect and the interaction between these formulation factors. The experimental condition has four independent variables (A, B, C, and D) and two dependent variables (X1 and X2) that were set to select the optimum formula by a Design-Expert software (version 8.2, Stat-Ease Inc., Minneapolis, MN). The optimized formula should have the least particle size (PS) and the highest zeta potential (ZP) as an absolute value (Table 2). Precirol ATO-5 and stearic acid were the tested solid lipids, while Labrafac and Tween 80 were the liquid lipid and surfactant, respectively. A total of 16 random runs were performed. Each run was performed in triplicate.

**Table 2.** 2^4^ full factorial design variables and levels of NLC-cinnamon oil.

Type of variables	Levels	
*Factors (independent variables)*	Lower limit	Upper limit
A: type of solid lipid	Precirol	Stearic acid
B: ratio of solid to liquid lipid	1:1 (min)	5:1 (max)
C: total lipids (%w/w)	10 (min)	20 (max)
D: surfactant concentration (%w/w)	0.5 (min)	3 (max)
*Responses (dependent variables)*	Desirability constraints	
X1: particle size (nm)	Minimize	
X2: zeta potential (mV)	Maximize (as an absolute value)	

### 2.5. Physicochemical characterization of NLC

#### 2.5.1. Particle size, zeta potential, and polydispersity index measurement

Mean PS, ZP, and polydispersity index (PDI) of the formulations were determined by Zetasizer Nano ZS90 (Malvern Instruments Ltd., Malvern, UK) at 25 °C at 90° angle. All samples were diluted with deionized water before analysis to reach a suitable scattering intensity. Each determination was performed in triplicates.

#### 2.5.2. Transmission electron microscopy (TEM)

Envisioning of the nanoparticle shape and surface characteristics of the prepared NLCs were performed using TEM (Joel JEM 1230, Tokyo, Japan). The diluted dispersion is negatively stained using a saturated solution of uranyl acetate for 5 min, followed by loading the stained formulation on a copper grid. A drying step of the copper grid lasted for 10 min at 25 °C followed by examination under the transmission electron microscope.

#### 2.5.3. Fourier transform infrared (FT-IR) spectroscopy

Functional groups of the pure cinnamon oil, stearic acid, Labrafac, Tween 80, and the optimized formulation were analyzed in the range of 4000–450 cm^−1^ by Cary 630 FTIR Spectrometer (Agilent, Santa Clara, CA), set on 74 scans at 4 cm^−1^ for each spectrum. All samples were analyzed with their original forms without further treatment.

#### 2.5.4. Entrapment efficiency and drug loading (DL%)

The entrapment efficiency (EE%) of the cinnamon oil in NLC was determined indirectly by measuring the concentration of free cinnamon oil in the NLC-cinnamon oil colloid using a Spin-pure filter (MWCO 10 kDa, Sera Care, Portland, ME). Accurately measured 100 µl NLC was diluted with 400 µl distilled water to remove unentrapped drugs that were deposited on the NLC surface. A total of 500 µl of this diluted sample mixture was transferred to the upper chamber of the filter tube which was then placed in a rotor and spun under ultracentrifugation at 20,000 rpm for 45 min at 4 °C. The filtrate collected in the bottom chamber was analyzed by UV-VIS spectrophotometry (Shimadzu UV-1800, Kyoto, Japan) to determine the unentrapped drug concentration. The detection wavelength was 293 nm. The EE% was calculated using the following equation. EE (%) = (total amount of drug in NLC – free drug/total amount of drug in NLC) × 100. All measurements were performed in triplicate.

### 2.6. NLC-cinnamon oil gel

#### 2.6.1. Preparation of NLC-cinnamon oil gel

NLC-cinnamon oil gel was prepared by a ‘cold’ method using thermosensitive Poloxamer 407 as a gelling agent (Wen et al., 2012). A concentration of 20% w/w NLC gel was selected according to a preliminary study of the gel concentration and desired formulation viscosity (data not shown). To prepare 20% w/w control and formulation gels, 10 g of Poloxamer 407 was dispersed in 50 g deionized water or NLC colloid and mechanical, slowly stirred at 100 rpm for two hours at room temperature until a homogeneous dispersion was obtained. The mixture was stored in the refrigerator at 4 °C for 24 hours to ensure complete dissolution due to its characteristic gelation phenomenon which is dependent on the sol–gel transition temperature (Tsol–gel). Below Tsol–gel, Poloxamer 407 aqueous solution is in solution form and turns into gel above this temperature (Dumortier et al., 2006).

#### 2.6.2. Spreadability

The spreadability test was determined using the same method reported by Ibrahim et al. (2019). A glass slide was first marked with a 1 cm diameter circle. Then, 0.1 g of the tested gel was transferred to the center of the circle and covered with another glass slide. This sandwiched glass piece was warmed on a hot surface with a temperature of 32 ± 0.84 °C. A metal balance weight (500 g) was topped over the glass cover for 5 min. The expanded circle diameter was recorded. The spreadability of the gel was determined by the difference in the diameters of circles due to the spreading of the gel. All measurements were performed in triplicate.

#### 2.6.3. pH measurement

The pH of the pure gel, blank NLC gel, and NLC-cinnamon oil gel was measured using a digital glass electrode-based pH meter (Schott Geräte, Mainz, Germany) standardized using pH 4 and pH 7 buffers before use. Each sample (0.5 g) was diluted with deionized water and dispersed uniformly before the measurement. In each measurement, the electrode was immersed into the sample for 10 min to reach a steady reading. The experiments were performed in triplicate at room temperature.

#### 2.6.4. Viscosity measurement

The apparent viscosities of the formulations were evaluated at 22.2 ± 0.5 °C using a Brookfield RV DV-II + pro viscometer equipped with a small sample adaptor. Each sample of 8 ml was introduced carefully into the adaptor chamber to avoid entrapping air bubbles. The selected spindle (S15) was lowered into the sample and rotated at various speeds from 50 to 200 rpm for at least 2 min before each reading was taken. Viscosity was calculated as a mean value of three measurements in centipoise (cP).

### 2.7. *In vitro* drug release

The release study was carried out using a shaking incubator (WiseCube^®^ WIS Precise Shaking Incubator, Wertheim, Germany) adjusted at 50 rpm (35 ± 0.5 °C) for five days. Each formulation (2 ml) was loaded into a dialysis membrane bag (MWCO 12–14 kDa, Spectra/por^®^ Spectrum Laboratories Inc., Rancho Dominguez, CA) with both ends closed by a thread and hanged on a beaker containing 400 ml release media of phosphate buffer pH 7.4 in which 30% absolute ethanol was added to ensure the sink condition for drug release. All membrane bags were immersed in the release media. The prepared beakers were positioned in the holders and gently shaken at a set speed in the incubator. One ml of released sample was withdrawn at the predetermined time intervals and replaced immediately by an equal volume of fresh, pre-warmed medium to maintain a constant receptor volume. Samples were suitably diluted and analyzed for the drug concentration by UV–Vis spectrophotometry at a wavelength of 293 nm. The amount of drug released was calculated according to the calibration curve equation (*Y* = 0.087*X* + 0.0234, *R*^2^ = 0.9907). The dilution caused by the replenish media of each sample was corrected using a relevant equation. Triplicate data were collected and their means were plotted against time (Raafat & El-Zahaby, 2020).

### 2.8. Determination of minimal inhibitory concentration (MIC)

The clinical isolates of *P. aeruginosa* strains were collected from burn and intensive care units in Alexandria University Main Hospital for this study and the patients were provided written informed consent. The isolation was approved by the ethical committee at the High Institute of Public Health, Alexandria University. Antimicrobial susceptibility testing was performed using the disc diffusion method, antibiotic discs tested were Meropenem (MEM, 10 μg), Ampicillin/sulbactam (SAM, 10/10 μg), Amoxicillin/Clavulanate (AMC, 10/10 μg), Ciprofloxacin (CIP, 5 μg), Cefepime (FEP, 30 μg), Cefoperazone (CFP, 75 μg), Cefotaxime (CTX, 30 μg), Ceftriaxone (CRO, 30 μg), Piperacillin (PIP, 100 μg), Imipenem (IPM, 10 μg), Gentamicin (CN, 10 μg), Tetracycline (TE, 30 μg), Levofloxacin (LEV, 5 μg), Trimethoprim/Sulfamethoxazole (TMP/SMX, 1.25/23.75 μg), Chloramphenicol (C, 30 μg), Amikacin (AMK, 30 μg), Aztreonam (ATM, 30 μg), Ceftazidime (CAZ, 30 μg) (Oxoid, Basingstoke, UK), and Colistin (CL, 10 μg) (Himedia, Mumbai, India) to all isolates. Obtained values were interpreted according to the Clinical and Laboratory Standards Institute into sensitive, intermediate, and resistant categories.

The antibiotic susceptibility profile for all strains is shown in Table 3. All *P. aeruginosa* isolates were resistant with varied resistance degrees for the tested antibiotics. The five isolated strains selected were multidrug-resistant and inoculated into the Luria-Bertani broth media with 30% glycerol and stored at −80 °C for later analysis.

**Table 3.** Antibiogram of used multidrug resistant *P. aeruginosa* strains.

Organisms	No. of isolates	The used antibiotics and percent of resistance pattern																		
		Gentamycin (GN)%	Amikacin (AK)%	Ampicillin/ Sulbactam (SAM)%	Amoxicillin/ Clavulanate (AMC)%	Cefepime (FEP)%	Cefoperazone (CFP)%	Cefotaxime (CTX)%	Ceftriaxone (CRO)%	Piperacillin (PIP)%	Imipenem (IPM)%	Meropenem (MEM)%	Ciprofloxacin (CIP)%	Levofloxacin (LEV)%	Trimethoprim/ Sulfamethoxazole (SXT)%	Aztreonam (ATM)%	Ceftazidime (CAZ)%	Chloramphenicol (C)%	Colistin (CL)%	Tetracycline (TE)%
*P. aeruginosa*	40	55.8	55.8	74.4	74.4	55.8	55.8	100	–	74.4	55.8	55.8	55.8	55.8	–	55.8	74.4	55.8	55.8	–

Broth microdilution method under standardized *in vitro* conditions was used to measure the minimum inhibitory concentration (MIC) of the formulations using a 96-well microdilution tray (Grianer, Pleidelsheim, Germany) with twofold drug-dilution steps. The growth of bacteria was visually evaluated according to the clearness of the solution (Abozahra et al., 2020). The pure cinnamon oil, pure gel, NLC blank gel, NLC-cinnamon colloid, and NLC-cinnamon oil gel, 2.5 mg/ml of each was twofold serially diluted with sterile nutrient broth, and then, 200 µl of each sample was transferred to the well of the microtiter tray. Ten µl of diluted broth cultures of *Pseudomonas aeruginosa* adjusted the bacterial suspension turbidity reaching a final inoculum of 0.5 McFarland turbidity standard which was transferred into each well. The microtiter tray was then incubated in the darkness at 37 °C for 24 hours, followed by visual inspection for bacterial growth by turbidity. Inoculated and uninoculated wells containing samples free of nutrient broth were used as controls to check the effect of the broth on the growth of the organism and the sterility. MIC value is the lowest concentration of each tested sample that inhibits the visible growth of *P. aeruginosa* (Jorgensen & Ferraro, 2009; Limbago, 2019). These tests were performed in all five strains of clinical isolates.

### 2.9. *In vivo* burnt wound study

Animal models used in the evaluation of the formulation treatment efficacy were male Sprague-Dawley rats (6–8 weeks, 200–220 g) supplied by Animal Supply Unit, Pharos University in Alexandria, Alexandria, Egypt. The study protocol was approved by the Unit of Research Ethics on Animal Experimentation of Pharos University in Alexandria (file no. 3025/2019-10). Animal care in the facility complies with the ‘Principles of Laboratory Animal Care’ by the American Society for Medical Research and the ‘Guide for the Care and Use of Laboratory Animals’ by NIH (Bethesda, MD).

The animals were maintained under a standard laboratory-controlled environment with free access to food and water and kept in the same conditions for at least three days before the experiment. The hot water method was used to produce burn injury (Chang et al., 2020). A total of 15 rats were divided into five groups with three rats in each group. Group 1 received no treatment (negative control), group 2 received cinnamon oil dispersed in inert carrier olive oil, group 3 received NLC-cinnamon colloid, group 4 received NLC blank gel, and group 5 received NLC-cinnamon oil gel. The dorsal hairs of all rats were shaved by clipping and disinfected the shaved area with 70% alcohol one day before the experiment. Animals were weighed and anesthetized with ketamine and xylazine before the burning procedure. A rubber sheet with a 2 cm circle opening was covered on the shaved dorsal skin. Rats were carried with the dorsal side down and immersed the exposed circular skin part into boiling water underneath for 10 s to induce a third-degree burn (Selcuk et al., 2012). Thereafter, a suspension containing approximately 7.5 × 10^5^ colony forming units/ml (CFU/ml) *P. aeruginosa* was inoculated through an injection under the wound area. Animals were kept individually in separate cages and fed with a standard rat diet and water for two days before starting the treatment.

Each tested formulation containing 7 mg of cinnamon oil was calculated for application amount in each group and applied onto the induced infected skin area of the rats once daily for six days. Bodyweight and burn edge contraction by the circle wound diameter were measured daily before the application of the next dose. Wound contraction was expressed as a reduction in the percentage of original wound diameter using the following equation (Tavares Pereira et al., 2012):

Wound contraction on day X (%) = [(area on day (−2) – area on day X)/area on day (−2)] × 100

All animals were euthanized by cervical dislocation at the end of the treatment.

### 2.10. Histological examination

The experimental burn skin area of each rat was dissected immediately after sacrifice. The specimens of the cutaneous tissues including epidermis, dermis, and subcutaneous portions, from all groups, were fixed in 10% neutral-buffered formalin, dehydrated with an ascending ethanol gradient, cleared using xylol, and embedded in paraffin to form formalin-fixed, paraffin-embedded blocks (FFPE). The blocks were cut into 5 µm sections then stained with hematoxylin and eosin (H&E). The sections were examined using different magnifications of the light microscope (Leica, Wetzlar, Germany) and photographed (Wen et al., 2014).

### 2.11. Statistical analysis

Design-Expert Program 8.2 software was used to generate a factorial design experiment and ANOVA was conducted to determine the significance of the factors and their interactions. For multiple comparisons, one-way analysis of variance (ANOVA) followed by post hoc Bonferroni’s test when comparing all pairs or Dunnett’s test when comparing pairs with control, with confidence degree 95% (*p* < .05) (GraphPad Prism 7, La Jolla, CA). All data were expressed as the mean of three experiments ± SD.

## 3. Results and discussion

### 3.1. Optimization of formulation by factorial design experiment

The formed NLC-cinnamon oil formulations were mostly white opaque colloids with various viscosities depending on the studied factors. Preliminary experimental trials were performed (data not shown) at first to select the factors as well as their levels. Full factorial design and statistical analysis were obtained using Design-Expert^®^ software. The predicted *R*^2^ of 0.5279 for PS and 0.4882 for ZP is in reasonable agreement with the adjusted *R*^2^ of 0.6998 for PS and 0.6746 for ZP, respectively. This means both *R*^2^ values are rationally matched (Abd-Elsalam et al., 2018). The factors and responses to the 16 random factorial runs are shown in Table 4. The optimized formulation generated by the program with desirability 0.892 showed the following responses: 108.48 ± 6.35 nm of PS, −37.36 ± 4.01 mV of ZP, solid:liquid lipid ratio of 4.5:1, total lipid concentration of 15.25% w/v, Tween 80 of 2.5% w/v, and stearic acid as solid lipid.

**Table 4.** The factorial design formulation matrix (*n* = 3).

Random run	Factor 1 (A) Types of solid lipid	Factor 2 (B) Solid:liquid lipid ratio	Factor 3 (C) Total lipid concentration % w/v	Factor 4 (D) Surfactant concentration % w/v	Response 1 (X1) Particle size, nm	Response 2 (X2) Zeta potential, mV
1	Stearic acid	5:1	10.00	3.00	120.00 ± 3.522	−37.60 ± 3.66
2	Precirol	1:1	10.00	0.50	212.70 ± 12.08	−25.20 ± 2.71
3	Precirol	5:1	10.00	3.00	677.10 ± 80.86	−38.70 ± 6.33
4	Stearic acid	5:1	10.00	0.50	130.30 ± 4.996	−34.70 ± 3.38
5	Stearic acid	1:1	20.00	0.50	173.60 ± 8.795	−39.00 ± 4.02
6	Stearic acid	1:1	20.00	0.50	104.70 ± 3.584	−30.80 ± 4.31
7	Precirol	1:1	10.00	3.00	169.50 ± 1.041	−39.10 ± 6.00
8	Stearic acid	5:1	10.00	3.00	140.50 ± 0.473	−35.60 ± 3.24
9	Precirol	1:1	20.00	0.50	220.50 ± 3.635	−35.20 ± 5.12
10	Stearic acid	5:1	20.00	3.00	109.70 ± 1.250	−43.10 ± 7.06
11	Precirol	5:1	20.00	0.50	157.20 ± 5.36	−23.80 ± 1.96
12	Precirol	1:1	20.00	3.00	236.00 ± 1.803	−34.60 ± 2.68
13	Precirol	5:1	20.00	0.50	141.50 ± 2.650	−11.30 ± 3.99
14	Stearic acid	1:1	10.00	0.50	172.30 ± 2.804	−43.50 ± 6.39
15	Precirol	3:1	15.00	3.00	136.50 ± 0.101	−35.50 ± 3.87
16	Stearic acid	5:1	20.00	3.00	113.60 ± 1.589	−30.30 ± 2.07

#### 3.1.1. Effect of formulation variables on particle size

**Figure 1.**
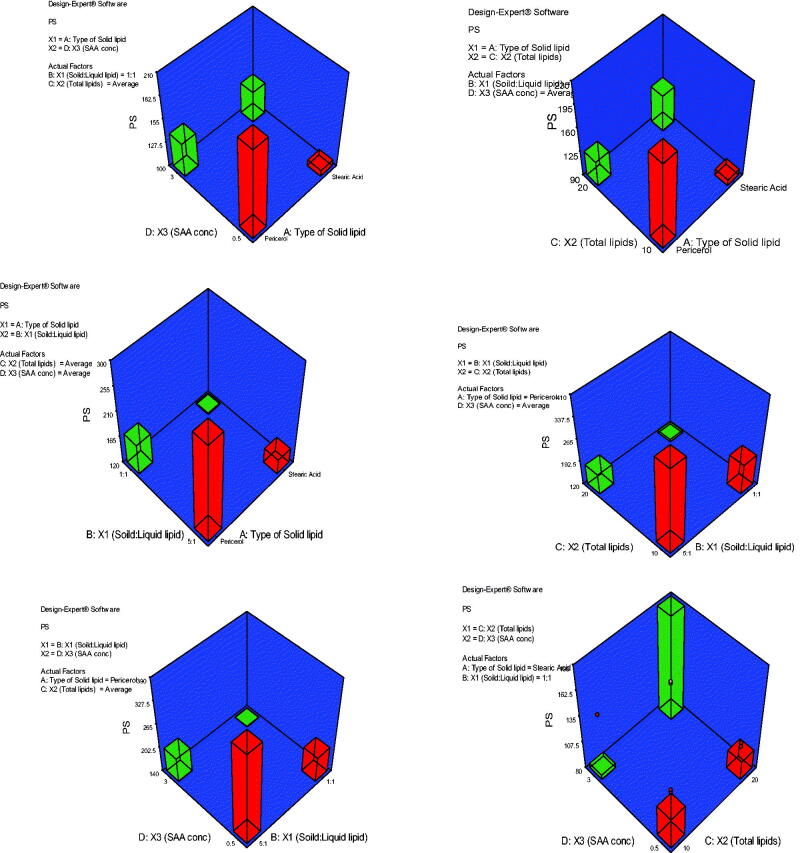
3D plot model graphs demonstrating the effect of factors. (A) Type of solid lipid, (B) solid:liquid lipid ratio, (C) total lipid content, and (D) SAA concentration on mean particle size (nm).

Optimizing the PS of the topically applied NLC is one of the important factors to be considered during formulation development. A nano-sized system can increase the probability of better and deeper drug penetration into skin strata (Abd-Elsalam et al., 2018). The effect of the formulation variables studied in the current research, including the type of solid lipid (A), solid:liquid lipid ratio (B), total lipid content (C), and surfactant concentration (D) on the mean NLC PS are illustrated in Figure 1. Stearic acid generated NLC with smaller PS than Precirol. The ANOVA data obtained from Design-Expert^®^ software for NLC PS statistical analysis showed that type of solid lipid (A) had a significant effect on the mean PS with stearic acid better than Precirol^®^ in decreasing the PS (*p* < .05). Compared to Precirol^®^, stearic acid has a much shorter carbon chain length which may permit the accommodation of a greater amount of active ingredient due to more space availability as suggested by Mahant et al. (2018). On the other hand, solid:liquid lipid ratios also showed a significant effect on NLC mean PS (*p* < .05), where the smallest PS was found in the 1:1 ratio. This result was in agreement with a study reported by Sathe et al. (2019) where the PS of NLC prepared using solid lipid (Precirol^®^ ATO 5) and liquid lipid Labrafac^TM^ PG had decreased from 321.06 ± 7.73 nm to 280 ± 15.583 nm when they used ratios of 2:1 and 1:1, respectively. Furthermore, a significant effect of total lipid concentration on NLC PS was also found (*p* < .05). PS decreased upon increasing the total lipid concentration from 10 to 20%. Higher lipid amount leads to the higher viscosity of NLC surface in which Labrafac^TM^ may tend to cause immense disorder in the crystal lattice due to its high HLB of 10, resulting in spontaneous emulsification in the presence of surfactant and yielding smaller PS. Our results came in agreement with those found by Gaba et al. (2015), where the PS decreased when they increased the concentration of total lipid in their NLC system. One has to consider the interactions between all factors and the materials used in the formulation. The PS of the formed NLC in this study decreased when surfactants concentration increased from 0.5 to 3%. It is also reported that NLC of lower PS can be obtained using high surfactant concentration (Pardeike et al., 2011). Higher surfactant concentration means reduced surface tension and consequently smaller size particles can be formed (Uprit et al., 2013). Overall, total lipid and surfactant concentrations had more profound effects on the PS reduction.

#### 3.1.2. Effect of formulation variables on zeta potential

**Figure 2.**
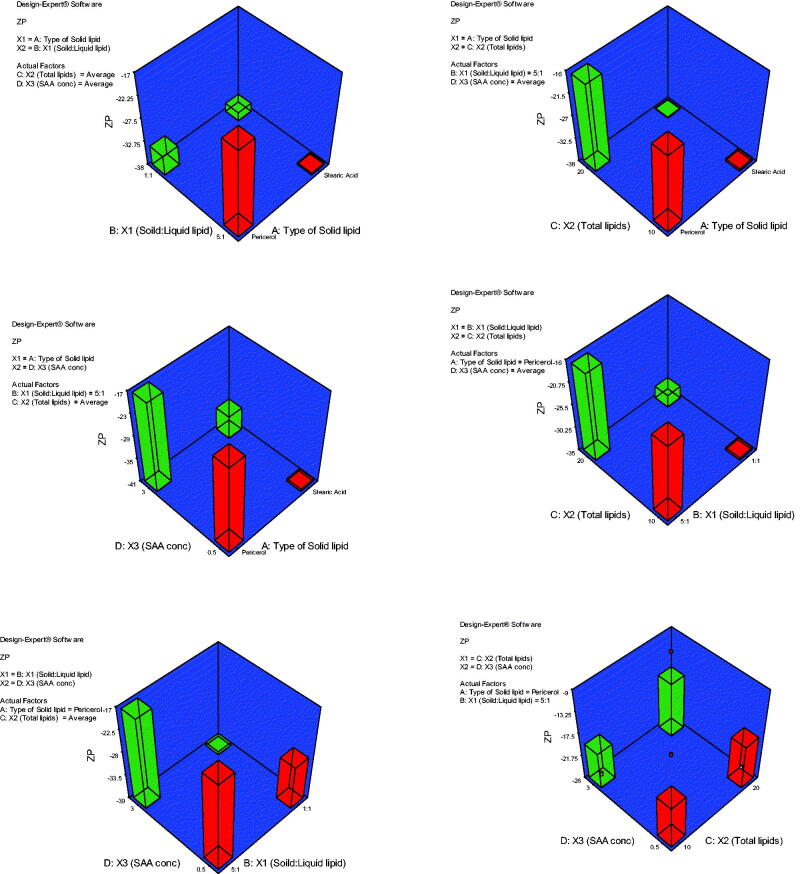
3D plot model graphs demonstrating the effect of factors. (A) Type of solid lipid, (B) solid:liquid lipid ratio, (C) total lipid content, and (D) SAA concentration on mean zeta potential (mV).

Figure 2 shows the effect of factors; A: type of solid lipid, B: solid:liquid lipid ratio, C: total lipid content, and D: SAA concentration on mean ZP (mV). The model generated for the ZP graphs had a *p* value of  < .0001 and an *F*-value of 7.43 which implied the significance of the model. The predicted *R*^2^ of 0.4882 is in reasonable agreement with the adjusted *R*^2^ of 0.6746. The ZPs of all the prepared NLCs were negative and ranged from −11.30 ± 3.99 to −43.10 ± 7.06 mV. The negative charge is attributed to the presence of either stearic acid or Precirol. Most of the NLCs had ZP values high enough to ensure physical stability due to both electrostatic repulsions and steric hindrance in the presence of long chains in the SAA structure (Vasanth et al., 2020). Besides, the PDI of these formulations ranged from 0.181 to 0.548 indicating homogeneity of nanoparticle size distribution.

### 3.2. Transmission electron microscopy

**Figure 3.**
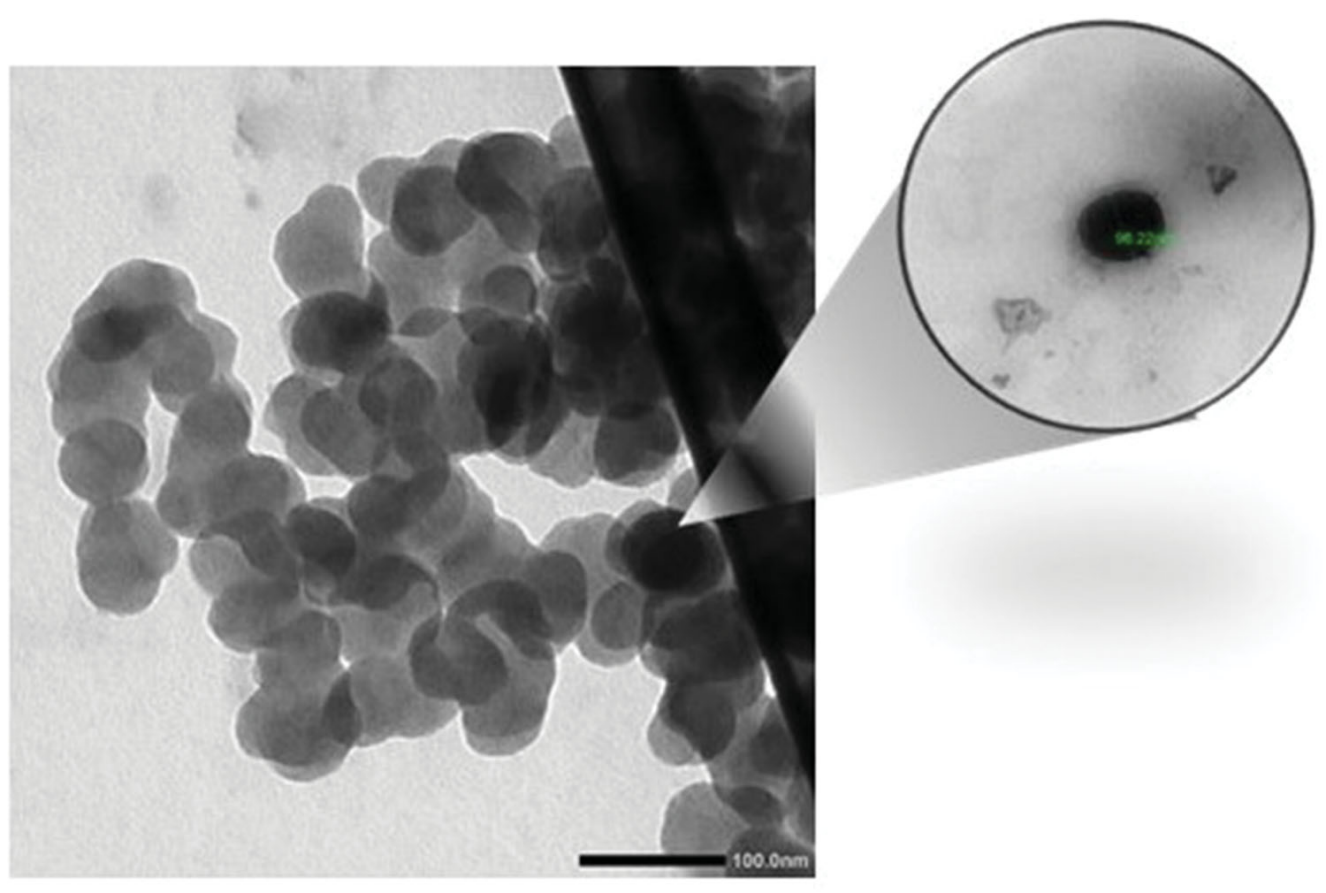
TEM image of NLC-cinnamon oil formulation.

TEM image confirmed that the prepared formulation contains smooth-surfaced spherical nanoparticles with an average size of 99.99 nm and uniform size distribution of the formed nanoparticles (Figure 3).

### 3.3. Fourier transform infrared spectroscopy

**Figure 4.**
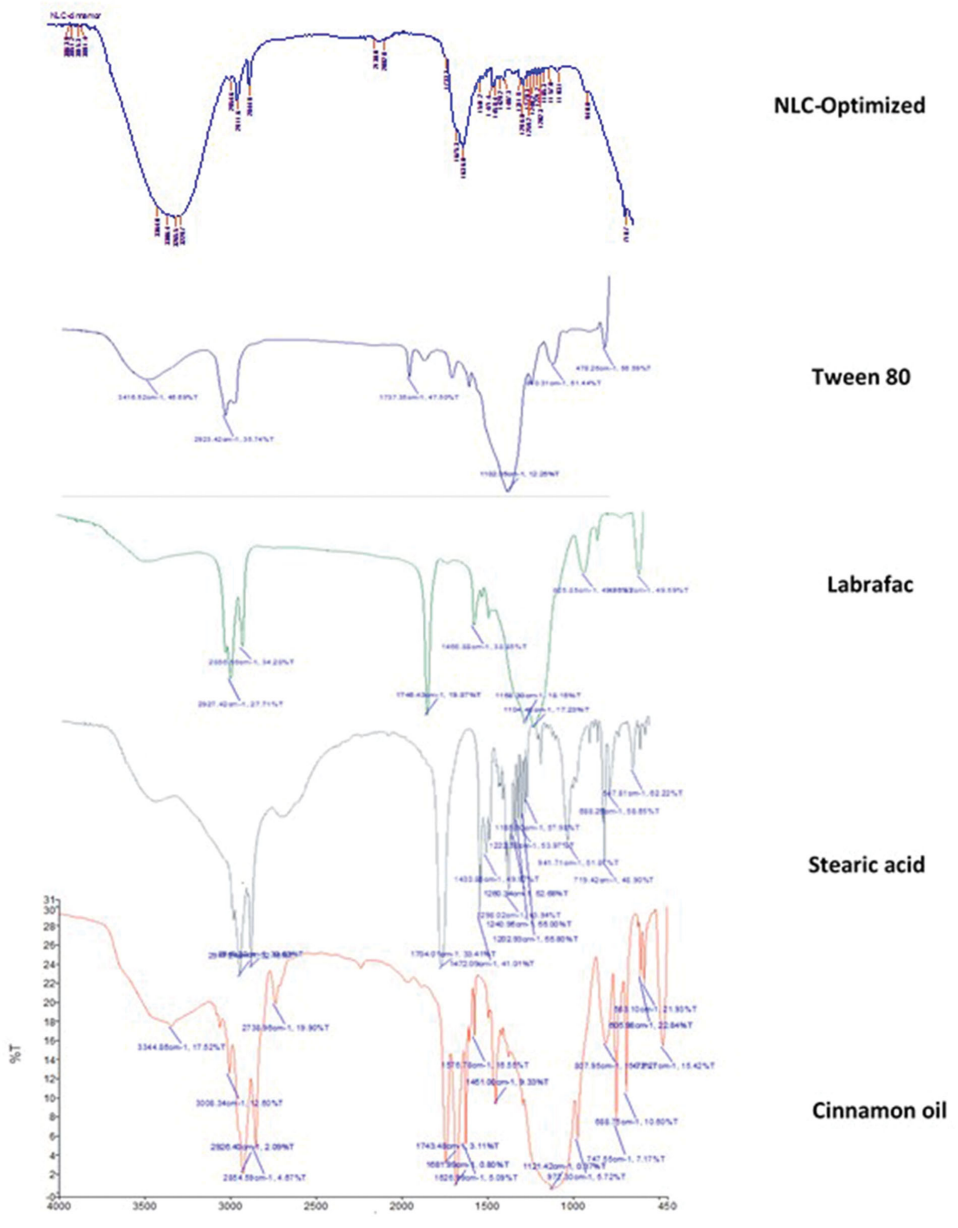
FTIR band spectra of cinnamon oil, stearic acid, Labrafac, Tween 80, and the chosen NLC-optimized formulation.

FTIR analysis based on molecular vibrations of band spectra is presented in Figure 4. The purity of the cinnamon oil from the prepared extract was confirmed with the theoretical frequency of the band (Boughendjioua et al., 2017) which showed the characteristic absorption peaks of the functional groups in chemical composition with aldehyde C=O stretching at 1743 and 1681 cm^−1^ and the strongest IR absorptions of carbonyl stretching at 1626 cm^−1^. These peaks corresponded to high levels of cinnamaldehyde and aldehydes of the sample. There was also the presence of unsaturated C=C stretching at 1576 and 1451 cm^−1^ and the aromatic =C–H stretching peak at 3008 cm^−1^ which was slightly higher than the –C–H stretching in alkanes at 2926 cm^−1^. Compared with pure cinnamon oil, the disappearance of these characteristic absorptions in the bands of spectrum of NLC-cinnamon oil indicated that cinnamon oil is well entrapped inside the nanocarriers. Also, the characteristic C–H asymmetric stretching peak of stearic acid at 2914 cm^−1^ and C–H symmetric stretching at absorbance peak at 2840 cm^−1^ (Amanda Velinna Fernandes et al., 2020) became much weaker in the absorption bands of NLC-cinnamon oil, indicating that stearic acid was well incorporated into nanoparticles. The broad O–H band around 3364–3229 cm^−1^ in NLC-cinnamon oil became shorter because the formulation was stabilized by Tween 80. The nanoformulation also showed hydrogen bond in hydrocarbon chain with bands at 2964 and 2844 cm^−1^ and carbonyl group at 1733 and 1675 cm^−1^. C=C stretch was also observed at 1541 and 1471 cm^−1^. Overall, the IR chart indicated a good formation of NLC nanoformulation which efficiently entrapped cinnamon oil with no excipient interaction.

### 3.4. Entrapment efficiency

Entrapment efficiency is one of the important parameters in developing nanoparticle delivery systems. High EE% indicates that the drug is successfully entrapped into the nanoparticle (Zhang et al., 2013). Cinnamon oil was very well entrapped in the nanoparticles with a high EE% of 95.39 ± 0.82% which confirmed the suitability and effectiveness of formulation design using NLC as nanocarriers for cinnamon oil.

### 3.5. Spreadability, pH, and viscosity of the NLC gel

Poloxamer 407 is a hydrophilic, nonionic copolymer with a center block of hydrophobic polypropylene oxide (PPO) and two hydrophilic polyethylene oxide (PEO) blocks on both sides. This thermosensitive hydrogel has bioadhesive properties and many applications in drug delivery showing the controllable sustained release of the drug from the formulation, such as parenteral, ophthalmic, nasal, vaginal, rectal, and topical applications (Fakhari et al., 2017). In preparation, Poloxamer 407 aqueous dispersion remains liquid under Tsol-gel due to self-assemble into micelles. Upon increasing the temperature, the polymer starts the gelling process due to the dehydration of the hydrophobic PPO blocks (Denisa Ficai, 2017).

The prepared NLC-cinnamon oil gel is a soft gel with milky color and pH 6.30 ± 1.02 which is suitable for topical application. Table 5 shows the comparison of spreadability and viscosity of the selected formulation and the control gels which exhibits a negative relation between the spreadability and viscosity. The lipid contents in the NLC increased the gel’s apparent viscosity. The viscosity of NLC-cinnamon oil gel was increased to 977.26 ± 22.6 cP which is about double the viscosity of the pure Poloxamer 407gel of 445.38 ± 28.89 cP.

**Table 5.** Comparison of the spreadability and viscosity of pure gel, blank NLC gel, and NLC-cinnamon oil gel (values are expressed as mean ± 1 SD, *n* = 3).

	Spreadability diameter (cm)	Viscosity (cP) (150 rpm, 22.2 ± 0.5 °C)
Pure gel	2.53 ± 0.59	445.38 ± 28.89
Blank NLC gel	1.82 ± 0.31	689.86 ± 49.47
NLC-cinnamon oil gel	1.46 ± 0.23	977.26 ± 54.24

### 3.6. *In vitro* drug release

**Figure 5.**
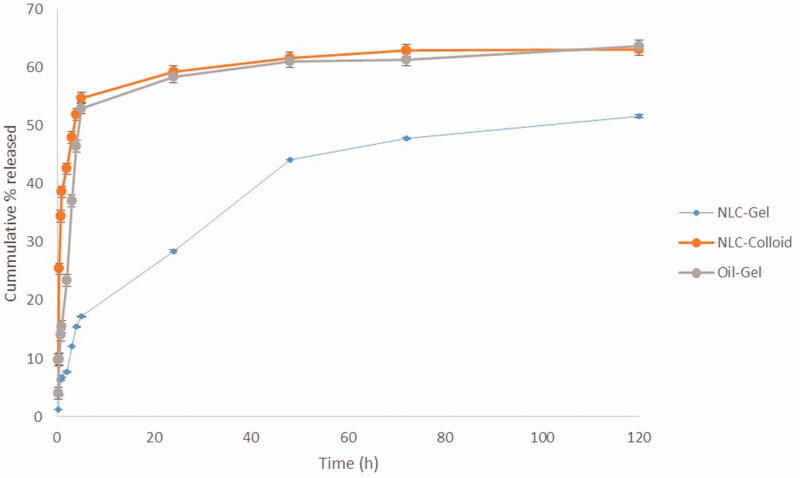
*In vitro* release of cinnamon oil from NLC colloid and gel compared to pure cinnamon oil gel in phosphate buffer pH 7.4 containing 30% absolute ethanol at 35 ± 0.5 °C (*n* = 3).

The percent cumulative cinnamon oil released from both NLC gel and colloidal systems were compared to the gel containing only the pure cinnamon oil. The drug release profile exhibited an initial burst release in all tested formulations in the first five hours, followed by a slow, sustained release up to five days; however, the NLC-cinnamon oil gel has less burst release effect compared to the NLC-cinnamon oil colloid and cinnamon oil gel, indicating gel containing NLC effectively entrapped cinnamon oil and controlled its release from the formulation (Figure 5). Herein, more control of the drug release was observed in the case of NLC-cinnamon oil gel in which the release pattern of cinnamon oil has achieved sustained manner. This result strongly confirmed an additive contributing effect of both NLC nanoparticles and gel base as barriers to the release of entrapped cinnamon oil. The difference calculated for NLC-cinnamon gel and cinnamon oil gel was found to be 45.84, indicating that the curves were not similar. Moreover, one-way ANOVA showed significant difference between the releases of cinnamon oil from these tested formulations. Further, post hoc Tukey’s multiple comparison tests found significant differences in drug release between the NLC-cinnamon oil gel and NLC-cinnamon colloid, and NLC-cinnamon oil gel and cinnamon oil gel (*p* < .05); however, no significant difference found in drug release between the NLC-colloid and cinnamon oil gel (*p*>.05) which further implied the synergistic effect in retarding the drug release.

By the 5th day, 51.6% of cinnamon oil was released from the NLC gel compared to 63.1% from the NLC colloid and 63.7% from the gel. To further explain this result, based on Fick’s law of diffusion and the Noyes–Whitney equation theories, experimental data were appropriately fitted into the Nernst–Brunner equation d*C*/d*t* = *DS* (*Cs* – *C*)/*Vh*, where, drug dissolution rate (d*C*/d*t*) is inversely proportional to path length (*h*). Herein, an increase in path length traversed by cinnamon oil through both NLC and gel until reaching the dissolution media will in turn decrease its release rate (Vasanth et al., 2020). Furthermore, we investigated the release mechanism of cinnamon oil from different formulations using various mathematical models, including zero-order, first-order, Higuchi, and Korsmeyer–Peppas models. The model with the highest coefficient of determination (*R*^2^) was considered to be the best fitting one. Cinnamon oil released from all test samples was found to follow the Korsmeyer–Peppas model (Table 6), with a best fit *R*^2^ value of 0.976, and *n* is equal to 0.409, 0141, and 0.234 for NLC-gel, NLC-colloid, and cinnamon oil, respectively. All the tested samples have a Fickian diffusion mechanism of drug release.

**Table 6.** Modeling of drug release from NLC-cinnamon gel, NLC-cinnamon colloid, and cinnamon oil-gel formulations.

	Zero-order	First-order	Higuchi	Korsmeyer–Peppas	
	*R* ^2^	*R* ^2^	*R* ^2^	*R* ^2^	*n*
NLC-gel	0.604	0.758	0.953	0.976	0.409
NLC-colloid	–4.000	–1.298	–1.590	0.760	0.141
Cinnamon oil-gel	–0.676	–0.022	0.297	0.794	0.234

### 3.7. Determination of minimal inhibitory concentration

Cinnamon bark oil has a wide variety of antimicrobial activities due to the presence of chemical compounds as cinnamaldehyde (the main constituent), eugenol, coumarin, and O-methoxycinnamaldehyde (Utchariyakiat et al., 2016). The antimicrobial effect of the cinnamon oil exerts probably through the action of these compounds. The concentration tested in this study were 0.0039%, 0.0078%, 0.0156%, 0.03125%, 0.0625%, 0.125%, 0.25%, and 0.5% w/v. Table 7 shows that NLC-cinnamon oil gel exhibits significant inhibitory activity against the test strain *P. aeruginosa* compared to all other formulations, with the lowest MIC of 312.50 μg/ml (ANOVA, *p* < .05). NLC-cinnamon oil colloid also showed a stronger anti-microbial effect than the cinnamon oil alone which was in agreement with the finding by Kim et al. (2015) that showed marked inhibition of biofilm formation, swarming motility, and the hemolytic activity of *P. aeruginosa* by cinnamon bark oil. The enhanced anti-microbial activity in NLC-cinnamon oil gel may also contribute to nanoparticles with large surface area and the lipophilic nature of the NLCs which enable better bacterial cell penetration and uptake (Osman et al., 2019). Both NLC-cinnamon colloid and NLC-cinnamon gel showed statistically significant MIC again *P. aeruginosa* compared to the cinnamon oil group (post hoc Bonferroni’s test, *p* < .05). The excipients of plain gel and NLC blank gel had no inhibitory effect against *P. aeruginosa* with the tested concentrations. The percentages decrease in MIC from NLC-cinnamon colloid and NLC-cinnamon gel compared to pure cinnamon oil was 75% and 87.5%, respectively.

**Table 7.** Comparison of MIC against five strains of *P. aeruginosa* in formulation and excipients.

	MIC (µg/ml) (*n* = 5)
Cinnamon oil	2250.00 ± 559.02
Plain gel	N/A
NLC blank gel	N/A
NLC-cinnamon oil colloid	562.00 ± 139.75
NLC-cinnamon oil gel	281.20 ± 69.99

### 3.8. *In vivo* burnt wound study

**Figure 6.**
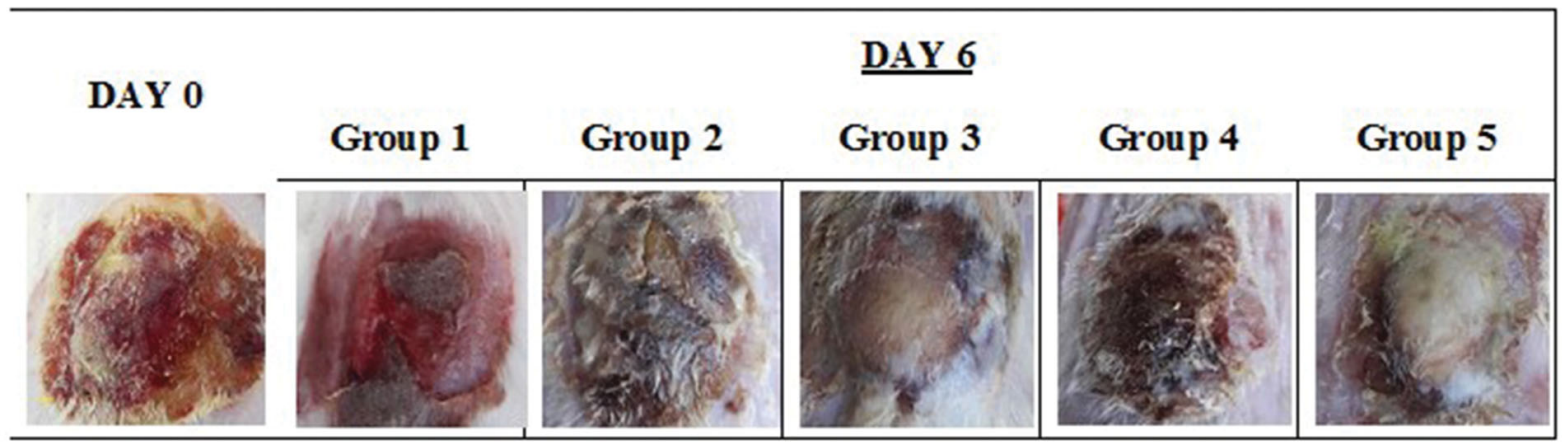
Images of original burn wound (day 0) and after treatment on day 6. Group 1: no treatment, group 2: cinnamon oil, group 3: NLC-cinnamon oil colloid, group 4: NLC blank gel, and group 5: NLC-cinnamon oil gel.

Visual assessment of the wound surface and edge contraction daily (Figure 6) showed that group 3 has similar healing progress as group 5 in the appearance of skin structure regrowth; however, group 5 presented more completed induration of firmly new skin growth and smooth wound edge with no signs of infection. The infected wound in group 1 (no treatment) appeared persistent erythematous infectious lesion during the treatment period with no improvement. Skin lesion in group 2, on the other hand, showed edematous and scarred surface with diffused wound edge which may be due to the irritation effect of the diluted cinnamon oil in the carrier oil directly applied on the open wound.

**Figure 7.**
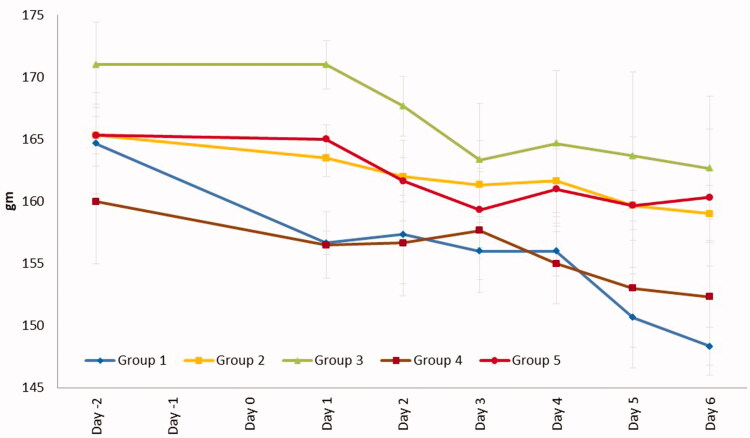
Weight changes during the treatment. Group 1: no treatment, group 2: cinnamon oil, group 3: NLC-cinnamon oil colloid, group 4: NLC blank gel, and group 5: NLC-cinnamon oil gel.

The reduction of body weight during the treatment period was highest in group 1 of 1.8498 g/day and lowest in group 5 of 0.7708 g/d (Figure 7). The percentage of wound contraction on day 6 (%) after completion of the treatment was in the order of group 5 (100.00%), group 3 (97.22%), group 2 (75.00%), group 4 (55.55%), and group 1 (30.55%). Treatment of NLC-cinnamon oil gel successfully healed the wound in six days.

**Figure 8.**
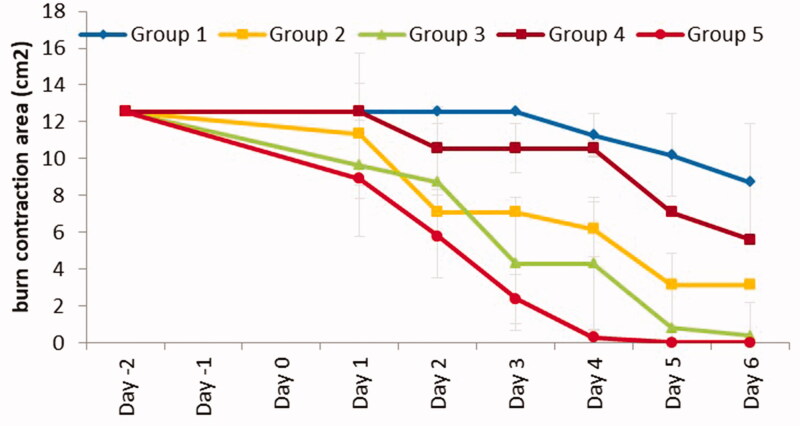
Burn area contraction (cm^2^) of tested groups. Group 1: no treatment, group 2: cinnamon oil, group 3: NLC-cinnamon oil colloid, group 4: NLC blank gel, and group 5: NLC-cinnamon oil gel (*p*<.0001).

The effectiveness of the tested formulation was additionally compared with control and excipient formulations by measuring the burn area contraction of the wound healing mechanism in rats (Abdullahi et al., 2014). Figure 8 shows that the burn contraction area was the smallest in group 5. Importantly, ANOVA (GraphPad Prism 8, La Jolla, CA) showed a significant difference between all groups (*p* < .0001). Further post hoc with Bonferroni’s pairwise comparisons indicated a significant reduction of burn contraction area between the control (group 1) and tested formulation (group 5) (*p* < .01), achieving the antibacterial outcome and accelerated wound healing. The rate of reduction in burn wound area was also highest in group 5 with 1.787 cm^2^/d and in the descending order of group 5 (1.787 cm^2^/d), group 3 (1.665 cm^2^/d), group 2 (1.2962 cm^2^/d), group 4 (0.8572 cm^2^/d), and group 1 (0.4548 cm^2^/d). Overall, NLC-cinnamon oil gel achieved significant completed wound healing on the burn wound infected by *P. aeruginosa* after a six-day treatment course.

### 3.9. Histological examination

**Figure 9.**
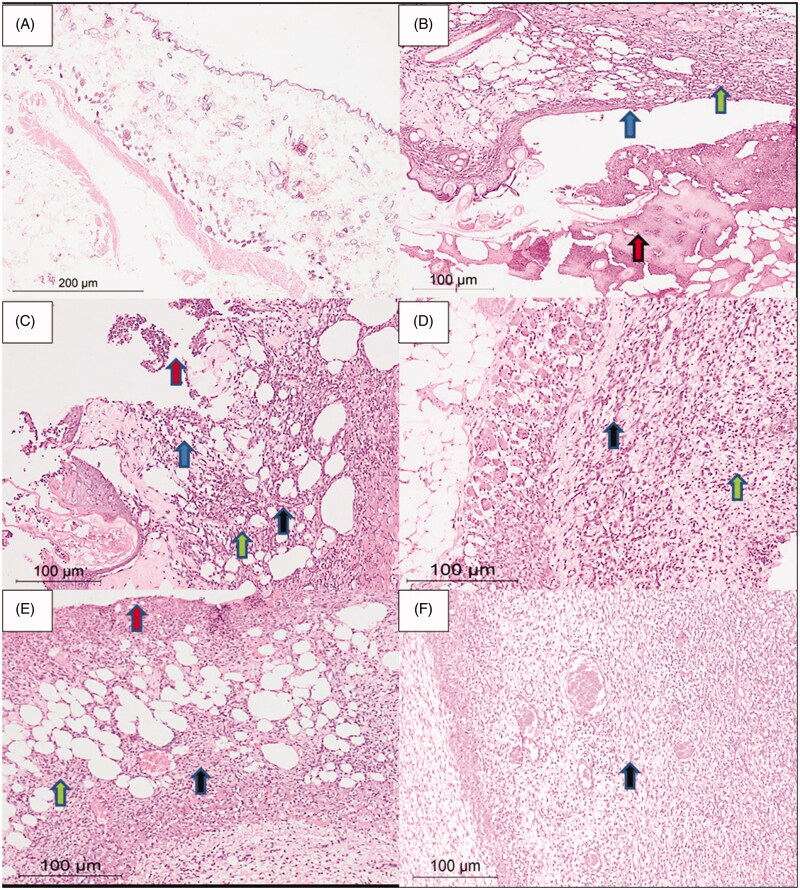
Rat skin samples after the six-day treatment. (A) The normal skin control group showed intact skin surface epithelium and normal dermal and subcutaneous tissues without inflammation (H&E, ×100). (B) Group 1 – no treatment: wide ulceration of the surface (blue arrow) covered by an excessive amount of fibrinoid material (red arrow). The dermis and the subcutaneous tissue show mixed acute and chronic inflammatory cells (green arrow), H&E ×200. (C) Group 2 – cinnamon oil gel: smaller skin ulcers (blue arrow) covered by fibrinoid material (red arrow). The dermis and subcutaneous tissue show inflammatory infiltrate (green arrow) and fibrosis (black arrow) H&E ×200. (D) Group 3 – NLC-cinnamon oil colloid: wide areas of granulation tissue (green arrow) admixed with fibrosis denoting the healing process (black arrow) H&E ×200. (E) Group 4 – NLC blank gel: focal ulcerated area covered by fibrinoid material (red arrow) with small areas of granulation tissue and fibrosis (black arrow) extending to the dermis and subcutaneous tissue with moderate mixed acute and chronic inflammatory infiltrate (green arrow), H&E ×200. (F) Group 5 – NLC-cinnamon oil gel: wide areas of granulation tissue and fibrosis occupying the whole dermis (black arrow), H&E ×200.

Figure 9 shows that the skin specimen from the normal control group presented typical cellular integrity with an intact surface epithelium, normal dermis, and subcutaneous tissue lacking any inflammatory infiltrate. The skin specimens from group 1 (without any treatment) showed a third-degree burn with wide ulceration of the surface epithelium covered by fibroid inflammatory exudate. The dermis part contained an inflammatory infiltrate formed of mixed acute and chronic inflammatory cells of neutrophils, lymphocytes, plasma cells, and histiocytes. The second group treated with cinnamon oil gel showed a smaller skin ulcer, compared to the first group, covered by fibrinoid material. The dermis and subcutaneous tissue showed mixed acute and chronic inflammatory cells. Group 3 (NLC-cinnamon colloid) showed a wide area of granulation tissue and fibrosis denoting the healing process with minor inflammatory infiltrate. The fourth group (NLC blank gel) showed skin ulceration covered by the fibrinoid material. Area of granulation tissue and fibrosis appeared in the dermis and subcutaneous tissue; however, less than group 3, while the inflammatory infiltrates were more than that of group 3. The fifth group (NLC-cinnamon gel) showed the most remarkable amount of fibrosis of the dermis and subcutaneous tissue with only a few inflammatory cells toward completion of healing.

## 4. Conclusions

Treatment for infected wounds should not only inhibit the causing microorganism but also promote the wound healing repairing process. The infected wound in experimental rats revealed that NLC-cinnamon oil gel significantly improved wound contraction percentage compared to the untreated and cinnamon oil groups by accelerated wound healing and antimicrobial effect. Our findings suggest that topical administration of NLC-cinnamon oil gel resulted in a significant antimicrobial activity and promotion of wound healing and skin regeneration. The studied nanoparticle-based cinnamon oil gel is a promising natural product against antibiotic-resistant strains of *Pseudomonas aeruginosa* in wound infection.
